# Knowledge, perception and practice towards oxytocin stability and quality: A qualitative study of stakeholders in three resource-limited countries

**DOI:** 10.1371/journal.pone.0203810

**Published:** 2018-09-25

**Authors:** Victoria L. Oliver, Peter A. Lambert, Kyu Kyu Than, Yasmin Mohamed, Stanley Luchters, Snigdha Verma, Ranjana Yadav, Vishwajeet Kumar, Alula M. Teklu, Moti Tolera, Abebaw Minaye, Michelle P. McIntosh

**Affiliations:** 1 Monash Institute of Pharmaceutical Sciences, Monash University, Melbourne, Victoria, Australia; 2 Burnet Institute, Melbourne, Victoria, Australia; 3 Department of Medicine, University of Melbourne, Melbourne, Victoria, Australia; 4 Department of Epidemiology and Preventive Medicine, Monash University, Melbourne, Victoria, Australia; 5 International Centre for Reproductive Health, Department of Obstetrics and Gynaecology, Ghent University, Ghent, Belgium; 6 Community Empowerment Lab, Lucknow, Uttar Pradesh, India; 7 MERQ Consultancy PLC, Addis Ababa, Ethiopia; 8 School of Public Health, Haramaya University, Harar, Ethiopia; 9 School of Psychology, Addis Ababa University, Addis Ababa, Ethiopia; Drake University College of Pharmacy and Heath Sciences, UNITED STATES

## Abstract

**Background:**

Oxytocin is the gold standard drug for the prevention of postpartum haemorrhage, but limitations in cold chain systems in resource-constrained settings can severely compromise the quality of oxytocin product available in these environments. This study investigated the perspectives and practices of stakeholders in low and lower-middle income countries towards oxytocin, its storage requirements and associated barriers, and the quality of product available.

**Methods:**

Qualitative inquiries were undertaken in Ethiopia, India and Myanmar, where data was collected through Focus Group Discussions (FGDs) and In-Depth Interviews (IDIs). A total of 12 FGDs and 106 IDIs were conducted with 158 healthcare providers (pharmacists, midwives, nurses, doctors and obstetricians) and 40 key informants (supply chain experts, program managers and policy-makers). Direct observations of oxytocin storage practices and cold chain resources were conducted at 51 healthcare facilities. Verbatim transcripts of FGDs and IDIs were translated to English and analysed according to a thematic content analysis framework.

**Findings:**

Stakeholder awareness of oxytocin heat sensitivity and the requirement for cold storage of the drug was widespread in Ethiopia but more limited in Myanmar and India. A consistent finding across all study regions was the significant barriers to maintaining a consistent cold chain, with the lack of refrigeration facilities and unreliability of electricity cited as major challenges. Perceptions of compromised oxytocin quality were expressed by some stakeholders in each country.

**Conclusion:**

Knowledge of the heat sensitivity of oxytocin and the potential impacts of inconsistent cold storage on product quality is not widespread amongst healthcare providers, policy makers and supply chain experts in Myanmar, Ethiopia and India. Targeted training and advocacy messages are warranted to emphasise the importance of cold storage to maintain oxytocin quality.

## Background

Postpartum haemorrhage (PPH) is estimated to cause approximately 20% of global pregnancy-related deaths with these deaths occurring overwhelmingly in low and lower-middle income countries (LMIC) [1]. Oxytocin is recognised by the World Health Organisation (WHO) as the gold standard therapy for the prevention of PPH and, when delivered as part active management of the third stage of labour, has been shown to reduce the risk of bleeding by 66% [2, 3]. However, oxytocin must be stored under cold (2–8°C) conditions in order to prevent drug degradation and maintain product quality [4]. Short term excursions outside of the cold chian are possible without significant loss of product quality, with exposure to 30°C for up to one month considered acceptable [4]. A number of oxytocin injection products are currently available which specify storage below 25°C, implying that, unlike those which recommend storage between 2–8°C, these products are heat stable and can be stored under ambient conditions. However, in order to maintain quality at higher storage temperatures, these products are marketed with a shorter shelf-life. In addition, these products are unsuitable for use in many LMIC, where hot climates and resource limitations will result in exposure to temperatures greater than 25°C if products are stored under ambient conditions [5].

Establishing and sustaining a consistent cold chain in resource-limited settings is challenging. Significant progress has been made towards maintaining stringent temperature-controlled storage of vaccines, predominantly through the establishment of the Expanded Program on Immunisation (EPI), a key strategy of which was to develop dedicated cold chain infrastructure and promote adequate knowledge and practices amongst policy makers and providers [6]. However, similar gains towards managing the storage of oxytocin and other temperature-sensitive medications have been less substantial. In recent years, advocacy bodies and development agencies have directed increasing attention to the importance of controlling oxytocin storage conditions to preserve product quality. The recent release of a joint statement by the WHO and UNICEF endorsing inclusion of oxytocin and other temperature sensitive drugs and medical products in the EPI cold chain is a significant step and should be strongly endorsed [7].

While policy dialogues around oxytocin storage have intensified amongst international health organisations, national guidelines in many LMICs often contain limited or varied information on oxytocin storage conditions. Oxytocin is listed on the essential medicines list for 110 out of 122 countries surveyed by the Reproductive Health Supplies Coalition [8], however, guidelines for drug storage are rarely included in these policy documents. In some countries, drug storage requirements are published in a national medicines formulary, however, guidelines for oxytocin storage are varied or limited. For example, storage of oxytocin at 2–8°C is recommended in the national formularies of Ethiopia [9] and Kenya [10], while the national formulary of India specifies storage below 30°C [11] and no guidance is provided in the formulary of Bangladesh [12], Nigeria [13], Nepal [14] or Zambia [15].

Over the past 25 years there have been several reports on oxytocin quality in LMIC, with the majority of publications released in the last five to seven years. The scale and scope of these studies range from collection and analysis of a handful of ampoules [4, 16], to large-scale post-marketing surveillance studies [17–20]. A recent systematic review has consolidated the evidence emerging from these studies and highlights that the quality of oxytocin ampoules available in LMICs is severely compromised, with more than half (57.5%) of ampoules collected in Africa and almost a quarter (23.3%) of ampoules in Asia failing to meet international quality specifications [21].

Despite the increasing body of literature emerging on oxytocin quality, less is known about the awareness of stakeholders involved in oxytocin supply, storage and use towards the importance of maintaining a consistent cold chain for oxytocin or their perceptions of the quality of product available. Therefore, a qualitative study was conducted in Myanmar, India and Ethiopia to explore the knowledge of healthcare providers, policy makers and supply chain experts about oxytocin stability and storage requirements. Their perceptions of oxytocin quality and experiences of barriers that exist to cold chain maintenance were also explored.

## Methods

Methods are reported according to the consolidated criteria for reporting qualitative research (COREQ) framework [22].

### Study design

An explorative qualitative study was conducted, where data was collected through Focus Group Discussions (FGDs), In-Depth Interviews (IDIs) and direct observations at healthcare facilities. FGDs were conducted to explore group norms and derive in-depth information through interactions between participants. IDIs were conducted to engage individuals with specialist knowledge and in circumstances where there was limited availability of participants (e.g. Ministry of Health stakeholders, specialist clinicians). Direct observations were conducted at healthcare facilities to triangulate responses from participants about oxytocin storage practices and cold chain infrastructure.

### Research team and reflexivity

In each country, a local principal investigator oversaw all aspects of the research, in collaboration with a project coordinator, who was responsible for assuring consistency of approach across territories. Data was collected by researchers with experience in qualitative research methods and a thorough understanding of the local context. All researchers were trained in the background and objectives of the study and given a refresher training on qualitative research methodology and research ethics. There was no pre-existing relationship between the participants and any researchers involved in data collection. Adjustments were made to vocabulary, tone and body language in order to minimise the impact of any power differentials between researchers and participants due to gender, social-economic status, or education.

This research was conducted as part of a broader study to understand the acceptability and feasibility of a novel heat-stable inhaled oxytocin product for the prevention of PPH. The findings presented here represent the needs assessment component, where the limitations of the currently available injectable oxytocin were explored (with particular focus on stakeholder awareness or perception of limitations). Additional topics that were explored with participants of this study included knowledge, attitudes and practices towards childbirth and PPH, perceived benefits and concerns regarding an inhaled oxytocin product and the operational feasibility of product introduction into the local health system. In order to minimise response bias, descriptions of the inhaled oxytocin product were kept brief during the informed consent process and only provided in detail after participants’ knowledge and perception of injectable oxytocin had been explored.

### Study settings

Research was conducted in Myanmar, India and Ethiopia, which were selected to represent the regions carrying a high burden of maternal mortality: South-East Asia, South Asia and Sub-Saharan Africa. Maternal health in Myanmar has improved significantly over the past decade, however, the country’s maternal health indicators remain amongst the poorest in the South-East Asian region. With a 2015 Maternal Mortality Ratio (MMR) of 178 deaths per 100,000 live births, Myanmar has failed to meet its Millennium Development Goal 5 target of 130 [23]. Likewise, in India, maternal health indicators still trail those of the developed world. There were an estimated 45,000 maternal deaths in India alone in 2015, the second largest number reported for any one country (behind Nigeria), accounting for 15% of the total number of maternal deaths worldwide [23]. In recent years Ethiopia has seen dramatic improvements in maternal health outcomes, with the MMR declining from 1,400 deaths per 100,000 live births in 2000 to 420 in 2013 [23]. However, with 11,000 maternal deaths in 2015, Ethiopia remains the fourth largest contributor to global maternal deaths, behind India, Nigeria and the Democratic Republic of Congo [23].

Recognising that there is significant within country variation in maternal health outcomes and indicators of health system functioning (including availability and reliability of cold chain facilities), research was conducted in urban and rural areas of each country to include settings where a large disparity in resource availability was expected. In Myanmar, research was conducted in urban and rural townships within Yangon region and in a rural township in Magwe region. In India, research was conducted in the state of Uttar Pradesh, where urban settings were selected from within Lucknow municipality and Sitapur district, and rural settings were selected from within Raebareili district. In Ethiopia, in addition to targeting urban and rural areas, research was conducted in a major region (Oromia) and two emerging regions (Gambella and Afar). Emerging regions (Afar, Somali, Benishangul-Gumuz and Gambella) of Ethiopia are well recognised to face particular health, economic and conflict-related hardships. Compared to the rest of the country, emerging regions score poorly on key maternal healthcare service indicators, such as overall readiness of facilities to provide routine delivery care (a composite score which comprises infrastructure, human resource and drug availability indicators) [24].

### Participant sampling

Participants of this study were healthcare providers involved in the use and storage of oxytocin including, nurses, midwives, doctors, obstetricians/gynaecologists and pharmacists. Key informants who played a role in oxytocin policy and supply were also engaged, including supply chain experts, Ministry of Health officials, and representatives from non-government organisations (NGOs) and professional associations. In order to explore knowledge and practice across the healthcare spectrum, healthcare providers were purposively sampled across a range of cadres (including community health volunteers through to obstetricians), from a range of health facility tiers (including health posts or sub-centres through to tertiary hospitals) in urban and rural areas of each country. Key informants were purposively sampled based on their position and experience and were typically engaged from major cities within each study region. Data were collected until it was felt that data saturation had been achieved. Across Ethiopia, Myanmar and India, a total of 158 healthcare providers and 40 key informants were engaged through 12 FGDs and 106 IDIs (see Table 1). Of the healthcare providers recruited, the predominant cadres directly involved in oxytocin use, storage and supply were those based at healthcare facilities (e.g. nurses, doctors, pharmacists). Community-based providers such as auxiliary midwives (Myanmar), Accredited Social Health Activists (India) and health extension workers (Ethiopia) were also recruited as they had received some level of training in intrapartum care and thus had an awareness of oxytocin, although they were not typically directly involved in the storage and supply of the drug. In Myanmar, the role of community-based providers (auxiliary midwives and midwives) in oxytocin use was found to vary considerably between villages and thus a relatively high number of FGDs were conducted with these providers in order to reach saturation of information. Direct observations were conducted at a range of health facility tiers involved in the provision of maternal health services, including health posts, health centres, hospitals, and private clinics. Direct observations were conducted at total of 51 healthcare facilities (see Table 2).

**Table 1:** Number of FGDs and IDIs conducted for each participant group in each country.

	FGDs (urban, rural)	IDIs (urban, rural)	Total participants (urban, rural)
**Myanmar**			
Auxiliary midwives	4 (1, 3)	-	29 (8, 21)
Midwives	3 (1, 2)	1 (0, 1)	27 (10, 17)
Nurses, health assistants	-	3 (1, 2)	3(1, 2)
Medical officers, township medical officers	-	3 (2, 1)	3 (2, 1)
Obstetricians	-	5 (5, 0)	5 (5, 0)
Pharmaceutical company representatives	-	2 (2, 0)	2 (2, 0)
UN agencies/NGO staff members	-	3 (3, 0)	3 (3, 0)
**Myanmar total**	**7 (2, 5)**	**17 (13, 4)**	**72 (31, 41)**
**India**			
Accredited Social Health Activist	1 (0, 1)	-	7 (0, 7)
Nurses	-	4 (2, 2)	4 (2, 2)
Doctors/obstetricians	-	5 (3, 2)	6 (4, 2)
Pharmacists	-	2 (2, 0)	2 (2, 0)
Supply chain experts	-	2 (2, 0)	2 (2, 0)
Government officials	-	2 (2, 0)	2 (2, 0)
Pharmaceutical company representatives	-	1 (1, 0)	1 (1, 0)
UN agencies/NGO staff members	-	2 (2, 0)	3 (3, 0)
**India total**	**1 (0, 1)**	**18 (14, 4)**	**27 (16, 11)**
**Ethiopia**			
Health extension workers	1 (1, 0)	8 (2, 6)	17 (11, 6)
Nurses, midwives, health officers	2 (2, 0)	22 (11, 11)	34 (23, 11)
Doctors/obstetricians	-	11 (9, 2)	11 (9, 2)
Pharmacists	-	10 (5, 5)	10 (5, 5)
Supply chain experts	-	8 (6, 2)	9 (7, 2)
Government officials	-	8 (5, 3)	9 (6, 3)
NGO and professional associations representatives	1 (0, 1)	4 (3, 1)	9 (3, 6)
**Ethiopia total**	**4 (3, 1)**	**71 (41, 30)**	**99 (64, 35)**
**Grand total**	**12 (5, 7)**	**106 (68, 38)**	**198 (111, 87)**	

**Table 2:** Number of direct observations conducted at healthcare facilities in each country.

Facility type	Obstetric care capability	Facilities observed (urban, rural)
**Myanmar**		
Sub-centre	Dispensary/ANC	1 (0, 1)
Rural health centre / maternal & child health centre	Dispensary/ANC	2 (0, 2)
Township hospital	CEmOC	2 (2, 0)
District, general or central hospital	CEmOC	3 (3, 0)
Private clinic / hospital	CEmOC	2 (2, 0)
**Myanmar total**		**10 (7, 3)**
**India**		
Urban health post	Dispensary/ANC	1 (1, 0)
Primary health centre	BEmOC	2 (0, 2)
Community health centre / maternal & child health centre	BEmOC	3 (2, 1)
Tertiary hospital	CEmOC	1 (1, 0)
Private clinic	CEmOC	1 (1, 0)
**India total**		**8 (5, 3)**
**Ethiopia**		
Health post	Dispensary/ANC	5 (0, 5)
Health centre	BEmOC	16 (9, 7)
Hospital	CEmOC	6 (4, 2)
Private or joint public / private	CEmOC	6 (4, 2)
**Ethiopia total**		**33 (17, 16)**
**Total**		**51 (29, 22)**

ANC = Antenatal clinic; CEmOC = comprehensive emergency obstetric care; BEmOC = basic emergency obstetric care.

### Data collection

Data was collected in Myanmar from June 2015 to February 2016, in India from October 2015 to February 2016, and in Ethiopia from April 2016 to August 2016. Written informed consent was obtained from all participants on the day of their involvement in the study, before commencing each FGD or IDI. Each FGD and IDI was conducted in a space that offered sufficient privacy and convenience for participants, such as private offices, community centres or private homes. FGDs consisted of between five and 12 participants. Typically, one participant at a time was engaged for IDIs, however, in some cases two respondents were interviewed together (at the suggestion of the participant and when it was felt by researchers that this approach would enhance data collection). During IDIs and FGDs, responses from participants were elicited by an experienced moderator using a semi-structured interview guide. The content of interview guides was consistent between study countries, however, the format and wording of guides was pilot-tested, adapted to suit the local context and translated into the local language. Guides were designed to be sufficiently prescriptive yet flexible such that they covered all necessary research domains, while allowing researchers to explore novel topics emerging during discussion.

Where consent was provided by participants, FGDs and IDIs were audio recorded using digital recorders. At least one note taker was present at each FGD and IDI to document non-verbal observations and keep a record of the content of the discussion (to complement audio recordings and/or serve as a back-up in case audio was not successfully recorded). FGDs and IDIs were drawn to a close at the point when researchers felt that discussion guides and emergent topics were adequately explored (or earlier if availability of the participants’ time was limited). FGDs lasted for between 40 and 120 minutes, while IDIs lasted for between 20 and 75 minutes. Direct observations were conducted to explore the practices of oxytocin storage and availability of cold storage infrastructure. Data from observations were recorded using a structured checklist.

### Data management and analysis

Audio recordings from FGDs and IDIs were transcribed verbatim in the local language, supplemented with written notes, and translated into English. English translations were verified through independent reverse translation. Thematic content analysis was applied, where transcripts from each country were coded by a team of three researchers using NVivo software (version 11). Each transcript was read by at least two analysts independently and open codes applied to units of meaning within words, phrases or paragraphs of text. Throughout the coding process, analysts met regularly to compare codes and develop a standardised coding framework, which was then applied to subsequent transcripts. At the conclusion of coding, intensive discussions were held among analysts to agree on a final thematic framework. This framework was discussed and finalised by all in-country research partners at an analysis workshop held at the conclusion of the study.

### Ethical considerations and approvals

This study involved the collection of qualitative data from individuals regarding their knowledge and practices towards oxytocin. While risks to participants were minimal, they included potential embarrassment or concern about admitting to knowledge or practices that contrast with global or national guidelines on oxytocin. Thus, measures were put in place to preserve the privacy, comfort and dignity of all participants. Written informed consent was obtained from all participants. Participants were assured that their data would be stored securely and accessible only to authorised researchers, and that no identifying information would be contained in any publications arising from the study. The design and procedures for research in all three countries were reviewed and approved by the Alfred Hospital Ethics Committee (Project 153/15) and ratified by the Monash University Human Research Ethics Committee (CF15/1701–2015000854). The Department of Medical Research approved research in Myanmar (48/Ethics 2015). The Community Empowerment Lab Institutional Ethics Committee approved research in India (CEL-IEC/2015003). The Scientific and Ethical Review Committee of the Ethiopian Public Health Institute approved research in Ethiopia (EPHI 6.13/269).

## Results

Analysis of data arising from this study are discussed in the context of three interrelated thematic categories: 1) the knowledge and practices of stakeholders towards the storage requirements for oxytocin; 2) perceptions of the quality or efficacy of the oxytocin product; and 3) descriptions of any constraints to reliable cold storage of oxytocin.

### Knowledge and practices around oxytocin storage

In Myanmar, oxytocin was not refrigerated at any of the healthcare facilities visited as part of the study. Providers also reported that oxytocin is not stored refrigerated in the public supply chain, either in storage warehouses or during distribution. Notably, oxytocin was stored under ambient conditions even when a functional refrigerator was available. The most common reason why ambient storage of oxytocin was practiced in health facilities was due to a belief in these to be the appropriate storage conditions for oxytocin (as was the case for the majority of nurses, midwives and auxiliary midwives from both urban and rural areas). Some doctors and obstetricians recognised the requirement for oxytocin to be refrigerated, but explained that oxytocin is stored under ambient conditions in line with historical practices. One obstetrician explained that as oxytocin was not refrigerated in the supply chain, and thus there was little point in keeping it cold in the facility. Another suggested that the need to refrigerate oxytocin was not a major concern in relation to the other healthcare challenges experienced.

We have to bear so many difficulties and using oxytocin without cold chain is not a big issue at all for developing countries

- Obstetrician, private clinic, Myanmar

Practices around cold storage of oxytocin in India were varied. In many public sector facilities oxytocin was stored at room temperature both in the pharmacy and in the labour ward, despite the availability of a functional refrigerator. This included primary healthcare centres and community healthcare centres in urban and rural areas. In addition, oxytocin was stored and transported under ambient conditions at a district level supply warehouse. In contrast, oxytocin was stored refrigerated in a community health centre, a tertiary hospital and a private clinic in an urban area. Ambient storage of oxytocin was reported to follow manufacturers’ guidelines, and all of the oxytocin brands observed at public healthcare facilities were labelled for storage ‘in a cool dry place’, leading providers to believe that storage at room temperature is sufficient.

[Storage instructions specify] only cool and dark place, that's all! Temperature maintenance is not given. Fifteen years back even I was not knowing that this (oxytocin) is kept in the refrigerator

- Obstetrician, private clinic, India

Almost all healthcare providers engaged in Ethiopia expressed their knowledge of the requirement for oxytocin to be stored refrigerated. This included health extension workers, nurses, midwives, pharmacists and obstetricians from both urban and rural areas across major and emerging regions. Many respondents explained that the oxytocin product will lose efficacy if it has not been kept refrigerated and therefore expressed their attitude that cold storage of the drug is not only a requirement in standard protocols, but highly important.

*Normally the medication (oxytocin) should be stored inside refrigerator from the production site up to the site of use…while it is stored out of refrigerator it reduces the effectiveness of medication (the effectiveness of oxytocin is reduced after storage outside of the refrigerator)*.

- Health extension worker, health post, Ethiopia

Accordingly, during IDIs, almost all healthcare providers reported that the drug is stored refrigerated at the facility at which they work. Many providers also reported that oxytocin is delivered refrigerated. However, data from direct observation at healthcare facilities indicated that the storage practice for oxytocin is more varied. Oxytocin was observed to be stored outside of the refrigerator in the labour wards of approximately one third (seven out of 21) of the healthcare facilities where this data was collected (five urban facilities and two rural). In some cases, observation of oxytocin storage conflicted with reports given by healthcare providers during IDIs. For example, at a hospital where an obstetrician and a nurse reported that oxytocin is stored refrigerated, only a small batch of ampoules (the daily supply) were kept in the refrigerator, whereas the rest of the stock was stored at room temperature. With the exception of this hospital, oxytocin was stored refrigerated when refrigerators were available at all other facilities where this data was collected.

### Perceptions of oxytocin quality

A prominent theme during discussions with some healthcare providers in Myanmar was the perception that the oxytocin injection used in practice has impaired effectiveness. Although these reports were not heard from nurses, midwives or auxiliary midwives, doctors and obstetricians described their experiences with unsatisfactory responses to oxytocin. To overcome perceived failings in oxytocin efficacy, high doses were reportedly administered for PPH treatment (up to 150 IU in contrast to WHO recommendations of 40 IU[3]).

*Maybe 20 unit or 40 unit or 80 unit or sometimes 100 unit. In the WHO recommendation is to give oxytocin, for example, 20 units in 1 litre of normal saline…but here we usually give in 500 cc of normal saline we add 20 unit, if nothing happen then we add 40 unit, sometimes we add up to 80 unit… So this is a problem. In our country the oxytocin dosage is very much higher than the recommended dose*.

- Obstetrician, private clinic, Myanmar

In addition, other uterotonics (such as misoprostol) were reportedly used in conjunction with oxytocin for prevention of PPH, a practice that was perceived by the respondent to be necessary due the sub-standard quality of the oxytocin product used. In most instances, the impaired efficacy of oxytocin was attributed to poor quality brands, where products from India and China were perceived as inferior. Some obstetricians speculated that improper storage also contributed to a decrease in the potency of the injectable product used.

Amongst healthcare providers engaged in India there was limited perception of the oxytocin product being of poor quality or having impaired effectiveness. Most obstetricians expressed their complete satisfaction with the quality and effectiveness of the oxytocin product they use. Others conceded that sometimes PPH occurs despite prophylactic administration of oxytocin but did not attribute this to a sign of compromised product quality. In contrast, an NGO program officer perceived there to be a decrease in the quality of oxytocin due to improper storage, in one instance leading to an adverse health outcome.

*I had noticed one case in the peak of summer, the oxytocin was kept in the labour room which was very, very hot and it did not work and the PPH actually happened*.

- Program officer, maternal health NGO, India

In Ethiopia, the majority of providers could not recall a time when oxytocin showed signs of impaired efficacy or quality when this question was put to them by researchers. This perception was similar for healthcare providers working in urban and rural areas and was particularly the case for nurses and midwives. However, a limited number of providers, mostly doctors, explained that oxytocin failed to stop bleeding after periods of inconsistent power supply, and reasoned that this was due to degradation of the drug.

*Six or seven months [ago], due to break up of refrigerator our midwife put oxytocin on shelf. And then it was administered to delivering mothers but couldn`t arrest bleeding. I think this is quality problem caused by keeping the product on the shelf*.

- Doctor, regional hospital, Ethiopia

Similarly, inadequate responses to oxytocin were described in relation to use of the drug for induction or augmentation of labour. Again, this was reasoned to be, in part, due to ineffective cold storage of the drug. Two obstetricians remarked on the problems of not knowing whether the quality of oxytocin has been compromised (due to degradation) and lamented on the paucity of published data in this area.

### Cold chain constraints

In both urban and rural areas of all study countries, all healthcare provider cadres expressed difficulties with maintaining a reliable cold chain. In Myanmar, cold storage facilities were available at hospitals in urban areas and used for the storage of vaccines. However, there were many descriptions of the constraints or unreliability of cold storage facilities. Reliability of electricity supply was identified as a problem due to power outages and fluctuation in voltage.

*In [township] the electricity is unstable and the range of the voltage also is not stable. Sometimes, it is very high and sometimes it is very low. In the industrial zone the power voltage is changing all the time*.

- Medical officer, township hospital, Myanmar

There was an absence of formal cold storage facilities at healthcare centres visited lower than the township hospital level. Refrigerators were not available at rural health centres and sub-centres. In these settings, items can be kept cold for short periods in vaccine transport carriers using ice bricks supplied from the nearest township hospital.

In India, cold storage facilities were available in the pharmacies of all health facilities visited that stocked oxytocin. However, refrigerators were not available in the labour wards of most facilities (with the exception of a private clinic and a tertiary hospital). Electricity supply was also reported to be intermittent or lacking, particularly in rural areas.

*Electricity here, it comes at 12:00 pm then it goes at 4:00 pm, again it will come at 10:00 pm and then again it will be available till morning, this is the system*.

- Pharmacist, primary health centre, India

One nurse suggested that recent privatisation of the electricity supply had resulted in inconsistencies, as hospitals must pay for their own electricity from constrained budgets. While facilities usually have a backup generator, some healthcare providers reported that the generator is sometimes out of fuel.

The majority of stakeholders engaged in Ethiopia suggested that maintaining appropriate cold storage of oxytocin is a problem in the supply chain and at service delivery points. This included a range of healthcare providers and key informants and was an equally prominent theme between the major region (Oromia) and emerging regions (Gambella and Afar). A key informant involved in pharmaceutical procurement suggested that challenges begin at import, as there is no dedicated cold storage room at the airport for pharmaceuticals. He explained that cold storage facilities are not available at all of the major public sector distribution warehouses and that there is limited space available in existing refrigerators.

*The same cold room is competing for different activities. Insulin is there. Oxytocin is there. The other vaccines are there. All those need the cold rooms and are competing for that limited space in those warehouses*.

- Supply chain manager, Ministry of Health, Ethiopia

The challenge of maintaining a consistent cold chain at healthcare facilities was largely attributed to the unreliability of the power supply and was a particularly prominent theme amongst healthcare providers from urban areas. These stakeholders suggested that cold chain constraints are most severe in rural areas but also faced in urban areas. Interestingly, discussions and interviews with providers from rural areas were more often weighted with suggestions that refrigerated storage is not a significant challenge, with most healthcare providers stating that back-up generators are available. However, some explained that these were either not functional, or only turned on for specific, high-need purposes (e.g. for surgical procedures rather than to power refrigerators), confirming that reliable cold storage is an issue across rural settings. Additional challenges are presented by the unreliability of refrigerators, particularly in emerging regions where high temperatures occurring during summer months were reported to compromise the ability of refrigerators to maintain temperatures below 8°C.

## Discussion

Through qualitative inquiry, this study explored the knowledge, perception and practices toward oxytocin of healthcare providers, supply chain experts and policy-makers in Myanmar, India and Ethiopia. Knowledge of the requirement for cold storage of oxytocin varied considerably amongst different stakeholders and between the three countries. While there was widespread understanding of the temperature sensitivity of oxytocin amongst all cadres of healthcare provider in Ethiopia, this knowledge was mixed amongst the providers engaged in Myanmar and India and often limited to doctors and specialists. Despite the widespread knowledge amongst stakeholders in Ethiopia, instructions for oxytocin storage conditions differ between key national policy documents. The Ethiopian National Formulary specifies storage at 2–8°C [9], while the Ministry of Health’s standard treatment guidelines for obstetrics recommends storage between 15–30°C [25]. Despite these contradictory guidelines, the Ethiopian Ministry of Health has demonstrated a strong commitment to ensuring oxytocin quality and improving PPH outcomes, which may have instilled an understanding of the importance of oxytocin cold storage across healthcare providers in the country. At the time of data collection, the Ministry of Health was in the process of establishing a dedicated task force with a mandate to reduce the country’s PPH mortality rate. In addition, the Ministry recently undertook and audit of oxytocin storage conditions and cold chain reliability [24] and supported a post-market surveillance study to investigate oxytocin quality in the public and private health system (publication in draft).

Previous studies have found that knowledge of oxytocin storage requirements varies between healthcare providers across many LMIC [26–28]. A qualitative study in India found that physicians, nurses and pharmacists had varying knowledge on how to store oxytocin, with some suggesting refrigerated storage is required, others suggesting that ambient temperature is sufficient [26]. Authors of this study reasoned that these findings illustrate that there is a lack of formal training of providers on the storage requirements for oxytocin. Review of national guidelines corroborate this theory, as recommendations differ considerably. The National Formulary released by the Government of India [11] indicates that oxytocin should be stored below 30°C, whereas guidelines released by the Federation of Obstetric and Gynaecological Societies of India [29] specify maintenance at 2–8°C with short term storage at 30°C as acceptable.

Practices around oxytocin storage largely mirrored provider knowledge of its storage requirements. However, in some cases, knowledge of the cold storage requirements for oxytocin did not translate into practice. For example, even the physicians in Myanmar who knew that oxytocin should be stored refrigerated were storing the drug under ambient conditions and explained that historical practices of storage were followed with limited concern for the implication on product quality. Similarly, in Ethiopia, widespread reports that cold storage of oxytocin was necessary and practiced were not always corroborated by findings from direct observation at healthcare facilities. While our studies indicated that oxytocin was stored refrigerated at approximately two third of facilities visited, a recent report released by the Ethiopian Ministry of Health found that oxytocin was stored refrigerated in over 90% of facilities [24]. The reason for this discrepancy in findings is unclear and warrants further investigation. More widely, several reports are emerging which show varied practices for oxytocin storage. A study conducted in India found that oxytocin was stored at room temperature in most facilities (93–100%) investigated in two districts in Uttar Pradesh, whereas 79–83% of facilities across two districts in Karnataka state were storing oxytocin under refrigerated conditions [18]. A study in Tanzania found that oxytocin was not refrigerated in 21 out of 29 hospitals investigated [30]. Likewise, oxytocin was typically found to be stored at room temperature in health centres in Sierra Leone [31]. Similar reports of ambient storage of oxytocin have emerged from Mongolia [32], Bangladesh [27] and Nepal [33].

A theme that was common to all countries in this study was the significant barriers to maintaining a consistent cold chain. Across all countries, this was highlighted to be a significant challenge in rural areas, Refrigeration facilities were not typically present at primary healthcare facilities in rural areas such as rural health centres and sub-centres (Myanmar) or health posts (Ethiopia). These findings are in line with published evidence. For example, a service provision assessment conducted in Myanmar highlighted that deficits in cold storage facilities were most significant in rural areas, where 44% of facilities have a cold chain system compared to 90% in urban areas [34]. In Ethiopia, a functional refrigerator was present at 77% of health centres according to a recent assessment of obstetric care services [24]. However, the findings of this study highlight that the effectiveness by which refrigerators can maintain temperatures between 2–8°C is sometimes in doubt, particularly in emerging regions with extremely hot climates. While and rural areas are commonly understood to face the most significant deficits in cold chain, in this study cold storage systems in urban areas were also reported to be unreliable. Inconsistencies in electricity supply were cited in this study as a major barrier to maintaining an effective cold chain across both urban and rural areas. Contingency resources such as back-up generators were sometimes reported to be inadequate for overcoming these interruptions in power supply, as these were in some cases either not functional (due to lack of fuel or damage) or not used for the purpose of powering refrigerators. The inadequacies of electricity supply have been reported in many LMICs [35–38]. A systematic review of the literature reporting on electricity access at health facilities in sub-Saharan Africa found that, on average, 74% of health facilities had access to electricity [39]. However only 28% of facilities reported electricity access to be reliable. Of the facilities where generator functionality was assessed, only a minority (10–29%) reported that the generator was functional at the time of the survey.

This study revealed interesting insights into provider perceptions on the quality of the oxytocin product. There were several providers in Myanmar who suggested that the oxytocin injection used in practice is not as ‘potent’ as it should be, citing the need to use high doses to effectively manage PPH as a potential indication of compromised product quality. While most providers attributed the substandard quality of oxytocin to the inferiority of the brand, it is possible that drug degradation may be a contributing factor given the widespread practice to store oxytocin under ambient conditions. Similarly, in Ethiopia, there were some providers who felt that the efficacy of oxytocin has been compromised at times and speculated that this was due to inadequate cold storage of the drug. It should be noted however, that provider speculations about impairments to the quality of oxytocin product were typically based on isolated instances of PPH occurring despite prophylactic administration of oxytocin. Rigorous clinical trials or retrospective analyses of large data sets would be required to determine whether poor quality of oxytocin correlates with higher rates of PPH in these settings. In Ethiopia, there are some published studies which cast doubt on the quality of the oxytocin product, as rates of failed induction following oxytocin administration are significantly higher in Ethiopia [40, 41] compared to high income countries [42, 43]. Authors of these studies hypothesised that interrupted cold storage of oxytocin may have led to drug degradation and thereby contributed to these high rates of failed induction.

### Strengths and limitations

A notable strength of this study is collection of qualitative insights into the storage practices and perception of oxytocin quality amongst different cadres of health care providers. This provides a nuanced understanding of stakeholder knowledge and perception around these subjects. Given that our aim was to seek in-depth insights, collection of data to quantitate statistically oxytocin storage practices was beyond the scope of this study, and findings have been drawn from published literature where available.

Investigation of a range of culturally, politically and environmentally diverse nations and sub-settings is a strength of this study and enables a comprehensive picture of the knowledge, attitudes and practice around oxytocin in these settings. However, it is understood that there is considerable intra- and inter-country variability that limit the direct extrapolation of the findings beyond the regions and countries investigated.

This study is a sub-set of a wider research program seeking to understand the barriers and enablers to the introduction of a heat-stable, inhaled formulation of oxytocin for the prevention of PPH in resource-limited settings. During FGDs and IDIs, researchers were careful to explore existing attitudes and practices towards oxytocin before providing an in-depth description of the inhaled oxytocin product. However, it is possible that participants expressing a knowledge and concern for the cold storage of oxytocin may have been influenced after being informed about the development of a heat stable formulation of oxytocin when consent was obtained. Direct observations of oxytocin storage practices were performed to triangulate reports from participants and limit the impact of this form of response bias.

## Conclusion

This study provides data to emphasise the challenges associated with maintaining a consistent and reliable cold chain in resource-constrained settings and describes the perception and experience of providers towards the quality of oxytocin products available in these contexts. These insights complement published quantitative data on compromised oxytocin quality and may help to drive action to address persistent oxytocin quality issues in low-resource settings. In this respect, adoption of heat stable uterotonics will help to address oxytocin quality issues caused by cold chain limitations. Additionally, the findings of this study highlight that knowledge of the heat sensitivity of oxytocin or cold chain management of the drug is not uniform within or across LMICs. Where deficits exist, training and education campaigns targeted towards healthcare providers are warranted to improve practices to help assure oxytocin quality.

## Supporting information

S1 File Coding framework and frequency of references for data from each country. (XLSX)

## References

[1] Say L, Chou D, Gemmill A, Tuncalp O, Moller AB, Daniels J, et al Global causes of maternal death: a WHO systematic analysis. Lancet Glob Health. 2014;2(6):e323–33. 10.1016/S2214-109X(14)70227-X . 25103301

[2] Begley CM, Gyte GM, Devane D, McGuire W, Weeks A. Active versus expectant management for women in the third stage of labour. Cochrane Database Syst Rev. 2011;(11):CD007412 Epub 2011/11/11. 10.1002/14651858.CD007412.pub3 . 22071837 PMC4026059

[3] WHO. WHO recommendation for the prevention and treatment of postpartum haemorrhage. Geneva: World Health Organisation, 2012. 23586122

[4] Hogerzeil HV, Walker GJA, deGoeje MJ. Stability of Injectable Oxytocics in Tropical Climates: Results of Field Surveys and Simulation Studies on Ergometrine, Methylergometrine and Oxytocin Geneva: WHO Action Program on Essential Drugs and Vaccines, 1993.

[5] World Health Organization. Stability testing of active pharmaceutical ingredients and fi nished pharmaceutical products: Annex 2. Geneva, Switzerland: World Health Organization, 2009.

[6] Lloyd J, Cheyne J. The origins of the vaccine cold chain and a glimpse of the future. Vaccine. 2017;35(17):2115–20. 10.1016/j.vaccine.2016.11.097 28364918

[7] World Health Organization, United Nations Children’s Fund. WHO/UNICEF joint statement: Temperature-sensitive health products in the Expanded Programme on Immunization cold chain World Health Organization—Expanded Programme on Immunization, United Nations Children’s Fund, 2015.

[8] Reproductive Health Supplies Coalition. EML Search: Database of Family Planning and Maternal Health Commodities in National Essential Medicines Lists New York, NY, United States: Management Sciences for Health,; 2013 [cited 2018 23/04/2018]. Available from: http://www.cecinfo.org/emlsearch/commodity/oxytocin/.

[9] Ethiopian Food Medicine and Healthcare Administration and Control Authority. Ethiopia Medicines Formulary In: Ethiopian Food Medicine and Healthcare Administration and Control Authority, editor. 2nd ed. Addis Ababa, Ethiopia 2013.

[10] Ministry of Medical Services. Kenya National Formulary for Primary Care Level. Ministry of Medical Services, 2008.

[11] Indian Pharmacopeia Commission. National Formulary of India. In: Welfare MoHF, editor. 4th ed. New Delhi, India2011.

[12] Ministry of Health & Family Welfare. Bangladesh National Formulary In: Directorate General of Drug Administration, editor. 4th ed. Bangladesh: Directorate General of Drug Administration,; 2015.

[13] Federal Ministry of Health of Nigeria. National Drug Formulary And Essential Drugs List Act. In: National Drug Formulary and Essential Drug List Review Committee, editor. 1989.

[14] Ministry of Health and Population. Nepalese National Formulary. In: Administration DoD, editor. 2nd ed. Nepal2010.

[15] Zambia Ministry of Health. Zambia National Formulary. In: Zambia National Formulary Committee, editor. Zambia2011.

[16] World Health Organization. Survey of the quality of medicines identified by the United Nations commission on life saving commodities for women and children. Geneva, Switzerland: World Health Organization Prequalification Team, 2015.

[17] Stanton C, Koski A, Cofie P, Mirzabagi E, Grady BL, Brooke S. Uterotonic drug quality: an assessment of the potency of injectable uterotonic drugs purchased by simulated clients in three districts in Ghana. BMJ Open. 2012;2(3). Epub 2012/05/05. 10.1136/bmjopen-2011-000431 ; PubMed Central PMCID: PMC3346944. 22556159 PMC3346944

[18] Stanton C, Nand D, Koski A, Mirzabagi E, Brooke S, Grady B, et al Accessibility and potency of uterotonic drugs purchased by simulated clients in four districts in India. BMC Pregnancy Childbirth. 2014;14(1):386 Epub 2014/11/14. 10.1186/PREACCEPT-1246552391229085 ; PubMed Central PMCID: PMC4240854. 25392131 PMC4240854

[19] Karikari-Boateng E. Post-market quality surveillance project: Maternal healthcare products (oxytocin and ergometrine) on the Ghanaian market Ghana Food and Drugs Authority (FDA) Laboratory Services Department & The Promoting the Quality of Medicines Program, 2013.

[20] Pribluda VS, Phanouvong S, Villadiego S, Rooslamiati I, Setiawati A, editors. Quality of Oxytocin Injections: a Case Study in Indonesia. Asia Regional Meeting on Interventions for Impact in Essential Obstetric and Newborn Care; 2012; Dhaka, Bangladesh.

[21] Torloni MR, Gomes Freitas C, Kartoglu UH, Metin Gulmezoglu A, Widmer M. Quality of oxytocin available in low- and middle-income countries: a systematic review of the literature. BJOG. 2016 10.1111/1471-0528.13998 . 27006180

[22] Tong A, Sainsbury P, Craig J. Consolidated criteria for reporting qualitative research (COREQ): a 32-item checklist for interviews and focus groups. Int J Qual Health Care. 2007;19(6):349–57. 10.1093/intqhc/mzm042 . 17872937

[23] WHO. Trends in Maternal Mortality: 1990 to 2015. WHO, 2015.

[24] Ethiopian Public Health Institute, Federal Ministry of Health, Columbia University. Ethiopian Emergency Obstetric and Newborn Care (EmONC) Assessment 2016. Addis Ababa, Ethiopia: Ethiopian Public Health Institute, Federal Ministry of Health, Columbia University, 2017.

[25] Ethiopia Ministry of Health. Management protocol on selected obstetrics topics. Addis Ababa, Ethiopia 2010.

[26] Deepak NN, Mirzabagi E, Koski A, Tripathi V. Knowledge, Attitudes, and Practices Related to Uterotonic Drugs during Childbirth in Karnataka, India: A Qualitative Research Study. PLoS One. 2013;8(4):e62801 Epub 2013/05/03. 10.1371/journal.pone.0062801 . 23638148 PMC3639256

[27] Bergeson-Lockwood J, Madsen EL, Bernstein J. Maternal Health Supplies in Bangladesh. Population Action International, 2010.

[28] Madsen EL, Bergeson-Lockwood J, Bernstein J. Maternal Health Supplies in Uganda. Washington DC, USA: Population Action International, 2010.

[29] Joshi BS, Gupte S, Ganguli I, Mane S, Singh U, Wagh G, et al. Consensus Statement for Prevention of postpartum haemorrhage (PPH). Mumbai, India: Federation of Obstetric and Gynaecological Societies of India (FOGSI), 2014 September, 2014. Report No.

[30] Mfinanga GS, Kimaro GD, Ngadaya E, Massawe S, Mtandu R, Shayo EH, et al Health facility-based Active Management of the Third Stage of Labor: findings from a national survey in Tanzania. Health Res Policy Syst. 2009;7:6 Epub 2009/04/18. 10.1186/1478-4505-7-6 ; PubMed Central PMCID: PMC2676279. 19371418 PMC2676279

[31] Natarajan A, Ahn R, Nelson BD, Eckardt M, Kamara J, Kargbo S, et al Use of prophylactic uterotonics during the third stage of labor: a survey of provider practices in community health facilities in Sierra Leone. BMC Pregnancy Childbirth. 2016;16:23 10.1186/s12884-016-0809-z ; PubMed Central PMCID: PMC4731897. 26821645 PMC4731897

[32] United Nations Population Fund (UNFPA), WHO. Joint UNFPA/WHO Mission in Collaboration with the Ministry Of Health to Review the Current Status of Access to a Core Set of Critical, Life-Saving Maternal/Reproductive Health Medicines in Mongolia Ulaanbaatar, Mongolia: UNFPA Country Office/Mongolia, 2009.

[33] Poudyal AK, Shrestha B, Onta SR. Availability and Use of Oxytocin in Health Facilities in Nepal. Journal of Institute of Medicine. 2014;36(1):3–8.

[34] Myanmar Ministry of Health, United Nations Population Fund (UNFPA). 2015 Health Facility Assessment for Reproductive Health Commodities and Services. Nay Pyi Taw, Myanmar: Department of Medical Research, Department of Public Health, Department of Medical Services, UNFPA, 2016.

[35] Ateudjieu J, Kenfack B, Nkontchou BW, Demanou M. Program on immunization and cold chain monitoring: the status in eight health districts in Cameroon. BMC research notes. 2013;6:101 10.1186/1756-0500-6-101 ; PubMed Central PMCID: PMC3630054. 23497720 PMC3630054

[36] Zambia Ministry of Health. Zambia Service Availability and Readiness Assessment Summary Report. Zambia2010.

[37] Malawi Ministry of Health. Malawi Service Provision Assessment 2013–14. Lilongwe, Malawi2014.

[38] Bangladesh Ministry of Health and Family Welfare. Bangladesh Health Facility Survey 2014. In: National Institute of Population Research and Training, Research AfCaP, editors. Dhaka, Bangladesh2016.

[39] Adair-Rohani H, Zukor K, Bonjour S, Wilburn S, Kuesel AC, Hebert R, et al Limited electricity access in health facilities of sub-Saharan Africa: a systematic review of data on electricity access, sources, and reliability. Glob Health Sci Pract. 2013;1(2):249–61. 10.9745/GHSP-D-13-00037 ; PubMed Central PMCID: PMC4168575. 25276537 PMC4168575

[40] Girma W, Tseadu F, Wolde M. Outcome of Induction and Associated Factors among Term and Post-Term Mothers Managed at Jimma University Specialized Hospital: A Two Years' Retrospective Analysis. Ethiop J Health Sci. 2016;26(2):121–30. ; PubMed Central PMCID: PMC4864341. 27222625 10.4314/ejhs.v26i2.6 PMC4864341

[41] Berhan Y, Dwivedi AD. Currently used oxytocin regimen outcome measures at term & postterm. I: Outcome indicators in relation to parity & indication for induction. Ethiop Med J. 2007;45(3):235–42. . 18330323

[42] Rouse DJ, Owen J, Hauth JC. Criteria for failed labor induction: prospective evaluation of a standardized protocol. Obstet Gynecol. 2000;96(5 Pt 1):671–7. . 11042299 10.1016/s0029-7844(00)01010-3

[43] Arulkumaran S, Gibb DM, TambyRaja RL, Heng SH, Ratnam SS. Failed induction of labour. Aust N Z J Obstet Gynaecol. 1985;25(3):190–3. . 3866556 10.1111/j.1479-828x.1985.tb00641.x

